# The Importance of Platelet Glycoside Residues in the Haemostasis of Patients with Immune Thrombocytopaenia

**DOI:** 10.3390/jcm10081661

**Published:** 2021-04-13

**Authors:** Andrés Ramírez-López, María Teresa Álvarez Román, Elena Monzón Manzano, Paula Acuña, Elena G. Arias-Salgado, Mónica Martín Salces, María Isabel Rivas Pollmar, Víctor Jiménez Yuste, Raul Justo Sanz, Sara García Barcenilla, Tamara Cebanu, Elena González Zorrilla, Nora V. Butta

**Affiliations:** 1 Hematology Unit, La Paz University Hospital-IdiPAZ, Paseo de la Castellana 261, 28046 Madrid, Spain; andres.ramirez1793@gmail.com (A.R.-L.); talvarezroman@gmail.com (M.T.Á.R.); elenamonzonmanzano@hotmail.com (E.M.M.); paulaacbu@gmail.com (P.A.); elenagas@hotmail.com (E.G.A.-S.); monicamsalces@gmail.com (M.M.S.); mirivas718@gmail.com (M.I.R.P.); vjyuste@gmail.com (V.J.Y.); rauljustosanz@gmail.com (R.J.S.); ehemostasia@gmail.com (S.G.B.); tamara.ce@hotmail.es (T.C.); elenaehemostasia@gmail.com (E.G.Z.); 2 Medicine Faculty, Autonomous University of Madrid, Arzobispo Morcillo 4, 28029 Madrid, Spain

**Keywords:** immune thrombocytopaenia, platelet apoptosis, sialic acid, platelet activation markers, glycoside residues

## Abstract

Loss of sialic acid from the carbohydrate side chains of platelet glycoproteins can affect platelet clearance, a proposed mechanism involved in the etiopathogenesis of immune thrombocytopaenia (ITP). We aimed to assess whether changes in platelet glycosylation in patients with ITP affected platelet counts, function, and apoptosis. This observational, prospective, and transversal study included 82 patients with chronic primary ITP and 115 healthy controls. We measured platelet activation markers and assayed platelet glycosylation and caspase activity, analysing samples using flow cytometry. Platelets from patients with ITP with a platelet count <30 × 10^3^/µL presented less sialic acid. Levels of α1,6-fucose (a glycan residue that can directly regulate antibody-dependent cellular cytotoxicity) and α-mannose (which can be recognised by mannose-binding-lectin and activate the complement pathway) were increased in the platelets from these patients. Platelet surface exposure of other glycoside residues due to sialic acid loss inversely correlated with platelet count and the ability to be activated. Moreover, loss of sialic acid induced the ingestion of platelets by human hepatome HepG2 cells. Changes in glycoside composition of glycoproteins on the platelets’ surface impaired their functional capacity and increased their apoptosis. These changes in platelet glycoside residues appeared to be related to ITP severity.

## 1. Introduction

Immune thrombocytopaenia (ITP) is an autoimmune disease characterised by a low platelet count (≤100 × 10^9^/L) due to platelet destruction and insufficient platelet production [1]. ITP is considered a rare disease (ORPHA 3002, OMIM 188030) that is diagnosed by ruling out other causes of thrombocytopaenia.

The initial event leading to antiplatelet autoimmunity remains unclear [2]; however, there is strong evidence that autoantibodies and autoreactive CD8+ cytotoxic T cells trigger enhanced platelet destruction and impair platelet production by megakaryocytes in the bone marrow [3]. ITP has been described as a deterioration of the regulatory compartment (regulatory T [Treg] and regulatory B [Breg] cells) of these patients’ immune system [4], along with a polarisation of the response towards T helper 1 (Th1) and Th17 cells. The abnormal T-cell function leads to the proliferation and differentiation of self-reactive B cells [5].

Many human autoimmune diseases are caused by acquired changes in glycanstructure or in their recognition by specific receptors. Platelets express highly glycosylated proteins on their surface that are involved in platelet haemostatic function and in the platelets’ interaction with other cells [6].

The role of glycans in platelet glycoproteins is poorly understood. Typically, glycans affect protein function by (1) guaranteeing proper protein folding, stability and solubility and (2) constituting key binding sites that are recognised by glycan-binding proteins, known as lectins. Glycoside residues are main players in cellular adhesion and intercellular communication [7]. The effects of N-linked and O-linked glycans on the stability of major platelets surface glycoproteins, including GPIb-IX-V, integrin αIIbβ3 (GPIIbIIIa) and GPVI, have been studied [8,9]. For example, disruption of the O-linked glycosylation mechanism in mice led to partial proteolysis of the glycoproteins and decreased GPIb-IX-V and αIIbβ3 functions, causing defective platelet activation and abnormal morphology, as well as excessive bleeding. The effect of desialylation on the surface glycoproteins of aged and refrigerated platelets has been more widely studied [10,11]. Loss of sialic acid induces the exposure of penultimate galactose that is recognised by hepatic Ashwell–Morell receptors, which leads to platelet clearance and triggers a feedback mechanism to increase platelet production through the hepatic expression of thrombopoietin [12].

Similarly, it has been reported that platelets from some patients with ITP have less sialic acid and that the patients’ thrombocytopaenia might improve through treatment with a neuraminidase inhibitor such as oseltamivir in combination with therapies to increase platelet production [13,14,15].

Nevertheless, there have been no in-depth analyses of glycans on the platelets of patients with ITP. The aim of our study was therefore to perform a comprehensive analysis of the platelet glycan repertoire to better understand their role in platelet function and in the development of ITP.

## 2. Materials and Methods

### 2.1. Study Design and Participants

This was an observational, prospective and transversal study that included patients with chronic primary ITP [1], who were stratified according to their platelet count (>30 × 10^3^/µL [65 patients] and <30 × 10^3^/µL [17 patients]). The study also included a healthy control group (115 participants) recruited from the blood donor section of the Haematology Unit of La Paz University Hospital. Inclusion period was from 10 January 2020 to 20 December 2020.

The study excluded patients with uncontrolled hypertension, hyperlipidaemia, peripheral or coronary artery diseases, abnormal hepatic or renal function, those undergoing therapy with platelet-active drugs and those who had undergone a transfusion within 15 days of the study. Regarding therapy not related to ITP, 3 patients were on antiretroviral therapy, 6 on atorvastatin, 1 on levothyroxine plus simvastatin, 3 on amitriptyline, 2 on lorazepam, 1 on furosemide plus amiodarone, 1 on hidroxicloroquine, 2 on metformin, 5 were receiving iron supplement, and most of the patients were on omeprazol.

The La Paz University Hospital Ethics Committee approved the experimental protocol (PI-3932), and the research study was conducted in compliance with the Helsinki Declaration and after receiving signed informed consent from the participants.

### 2.2. Collection and Preparation of Samples

We collected human peripheral blood samples in 3.8% sodium citrate and performed blood cell counts with a Coulter Ac.T Diff cell counter (Beckman Coulter, Madrid, Spain). We obtained platelet-rich plasma (PRP) by centrifuging the whole blood (150 g for 20 min at 23 °C). To obtain washed platelets, we collected the top two-thirds volume of the PRP and centrifuged it (650× g for 10 min at 23 °C) after adding acid-citrate-dextrose (1:10). The pellet was resuspended in an equal volume of HEPES buffer (10 mM HEPES, 145 mM sodium chloride, 5 mM potassium chloride and 1 mM magnesium sulfate, pH 7.4).

For the serum preparation, we collected peripheral blood in serum tubes (BD Vacutainer, Plymouth, UK) and separated it by centrifuging clotted blood (2500× g for 15 min at 23 °C). The plasma and serum aliquots were stored at −80 °C until analysis.

### 2.3. Determination of Platelet Activation Markers

We diluted the PRP 1:5 with HEPES buffer and incubated it with 100 µM of thrombin receptor-activating peptide 6 (TRAP, Bachem, Switzerland) or 20 µM of adenosine diphosphate (ADP, SIGMA, Madrid, Spain) at room temperature. Following incubation, we added fluorescein-isothiocyanate (FITC)-PAC1 (BD, Madrid, Spain), a monoclonal antibody (mAb) that recognises activated conformation of fibrinogen receptor, or FITC-labelled anti-human P-selectin mAb (BD Pharmingen, San Diego, CA, USA) or FITC-anti-CD63 mAb (Becton Dickinson, Madrid, Spain) for 15 min at room temperature in the dark.

We determined the surface expression of the fibrinogen receptor by labelling diluted PRP with phycoerythrin (PE)-mAbs against its αIIb (CD41, BioCytex, Marseille, France) and FITC-mAb against its β3 (Becton Dickinson) subunits. Surface expression of von Willebrand factor (VWF) receptor was determined using FITC-mAbs against its CD42a and CD42b subunits (BD Pharmingen, Madrid, Spain).

After incubation, all samples were diluted in PBS buffer for flow cytometry analysis with a FACScan flow cytometer (BD Biosciences, Madrid, Spain), and 10,000 events in the platelet region were acquired and analysed with BD CellQuest Pro™ software (BD Biosciences, Madrid, Spain).

### 2.4. Lectin Binding Studies

We incubated washed platelets (50 × 10^3^ platelets/μL) with FITC-labelled lectins (10 μg/mL, Vector Laboratories, Barcelona, Spain), as listed in Table 1, for 30 min at 37 °C and analysed them by flow cytometry.

### 2.5. Measurement of Apoptosis Markers in Platelets

We assessed the surface exposure of phosphatidylserine in washed platelets by measuring the binding of FITC-labelled annexin V (BD Pharmingen, Madrid, Spain). Briefly, washed platelets were resuspended in annexin V binding buffer (10 mM HEPES, 10 mM sodium hydroxide, 140 mM sodium chloride, 2.5 mM calcium chloride, pH 7.4) and labelled with FITC-annexin V. After a 15-min incubation period with either buffer or 1 µM ionomycin (Sigma, Madrid, Spain) at room temperature in the dark, the samples were analysed by flow cytometry.

To analyse active caspase-3, -8 or -9, we diluted PRP 10-fold with isotonic HEPES buffered saline with calcium ion (150 mM sodium chloride, 2 mM calcium chloride, 2 mM magnesium chloride, 2 mM HEPES, pH 7.4), 2 mM Gly-Pro-Arg-Pro (SIGMA, Madrid, Spain) and either FAM-DEVD-FMK, FAM-LETD-FMK or FAM-LEHD-FMK (Millipore, Madrid, Spain).

### 2.6. Neuraminidase Activity

We measured neuraminidase (NEU) activity in plasma and serum from the healthy controls and patients with ITP according to van der Wal et al. [16]. Briefly, we treated either plasma or serum with 75 µM of sodium acetate (pH 4.5), 0.1% Triton X-100 and 0.5 mM 2′-(4-methylumbelliferyl)-α-D-N-acetylneuraminic acid sodium salt hydrate (MUNANA, Merck, Madrid, Spain) in 96-well plates. We measured the fluorescence at beginning (time 0) and at 10, 15, 30, and 60 min after initiating the reaction (excitation λex = 365 nm, emission λem = 450 nm).

### 2.7. HepG2 Uptake of Platelets

Human HepG2 cells were grown in DMEM (Invitrogen, Madrid, Spain) supplemented with 10% heat-inactivated foetal calf serum (Lonza, Madrid, Spain) and penicillin/streptomycin at 37 °C and 5% carbon dioxide.

We seeded the HepG2 cells (1 × 10^5^/well) onto 24-well plates, allowed them to adhere for 24 h and then starved them for 30 min with serum-free media. The assay was initiated by adding platelets from healthy controls or patients with ITP (5 × 10^7^/well) previously labelled with 100 µM of CMFDA Cell Tracker (ThermoFisher Scientific, Madrid, Spain). HepG2 cells and platelets were incubated together for 45 min at 37 °C with gentle shaking. We washed the cultures three times and separated the HepG2 cells from the plates and from the surface-adhered platelets with a solution of trypsin/EDTA. After washing the HepG2 cells, we analysed them by flow cytometry according to their forward and side scatter features, and those that contained ingested platelets were identified by their fluorescence.

### 2.8. Statistical Analysis

We employed the Shapiro–Wilk test to assess the data distribution, and the results are presented as mean ± SD or median (p25–p75) depending on their distribution. We assessed differences between 2 groups using the 2-tailed unpaired Student’s *t*-test or the nonparametric Mann–Whitney U-test, as appropriate. To compare multiple groups, we performed a one-way analysis of variance or Kruskal–Wallis with Dunn’s multicomparison tests. The correlation analysis was performed using Pearson’s or Spearman’s test. We employed GraphPad Prism 5 software (GraphPad Software version 5.03) for all statistical analyses and set the significance at *p* ≤ 0.05.

## 3. Results

### 3.1. Features of Patients with Immune Thrombocytopaenia

Table 2 lists the characteristics of the patients with ITP.

**Figure 1:**
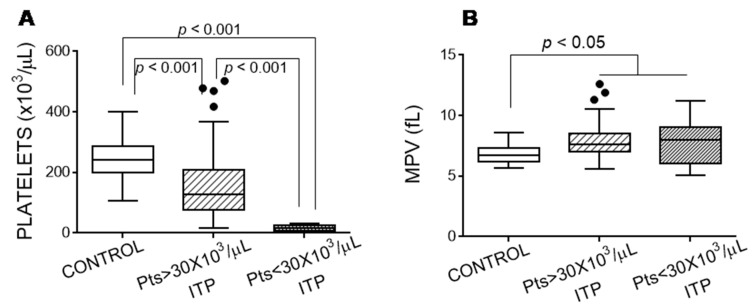
Platelet features in the patients with immune thrombocytopaenia (ITP). (**A**) Platelet count and (**B**) mean platelet volume (MPV). We performed Kruskal–Wallis and Dunn’s multiple comparison tests and considered a *p*-value of < 0.05 as significant.

Figure 1 indicates the platelet counts for the healthy controls and ITP groups, showing that the platelets from the patients with ITP had a higher mean platelet volume (MPV) than those from the controls (Figure 1). Moreover, the lowest platelet count was accompanied by the highest MPV (Spearman *ρ* = −0.562, *p* < 0.0001).

### 3.2. Exposure of Glycoside Residues on the Platelet Surface

**Figure 2:**
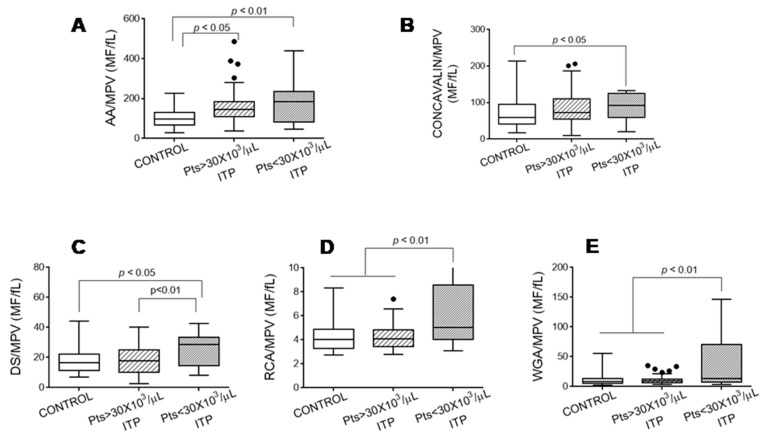
Lectin binding was determined in quiescent platelets from the controls and patients with immune thrombocytopaenia. Data are expressed as the ratio between the mean fluorescence of positive cells (MF) and mean platelet volume (MPV). The following lectins were tested: (**A**) Aleuria aurantia (AA), (**B**) Concanavalin A, (**C**) Datura stramonium (DS), (**D**) Ricinus communis agglutinin (RCA), and (**E**) Wheat germ agglutinin (WGA). We performed Kruskal–Wallis and Dunn’s multiple comparison tests and considered a *p*-value of <0.05 as significant.

Given that the platelets from the patients with ITP had a larger volume than those of the controls, we expressed the glycoside composition of the platelet surface as the ratio between the mean fluorescence of the binding of lectins and MPV. Figure 2 shows that the platelets from the patients with ITP had a different glycosylation pattern on their surface than the healthy controls, and this difference was more pronounced in the platelets from the patients with ITP with <30 × 10^3^ platelets/µL.

### 3.3. Platelet Activation Markers

**Figure 3:**
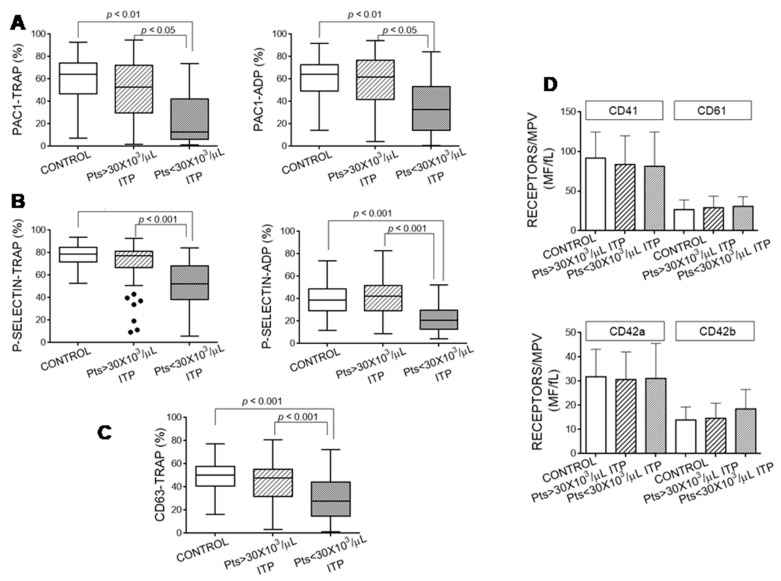
Platelet activation markers. Platelets from healthy controls and from patients with immune thrombocytopaenia (ITP) stimulated with thrombin receptor-activating peptide (TRAP) and ADP were incubated with fluorescein isothiocyanate (FITC)-PAC1 (**A**), FITC-anti-P-selectin monoclonal antibody (mAb) (**B**) or FITC-anti-CD63 mAb (**C**). The data in (**A**–**C**) are expressed as percentage of positive cells. (**D**) Fibrinogen receptor (subunits CD41 and CD61) and VWF receptor (subunits CD42a and CD42b) are expressed as the ratio between mean fluorescence (MF) and mean platelet volume (MPV). All samples were analysed by flow cytometry. We performed Kruskal–Wallis and Dunn’s multiple comparison tests and considered a *p*-value < 0.05 as significant.

We determined the platelets’ ability to be activated after agonist stimulation in our cohorts. Platelets from the patients with ITP with <30 × 10^3^ platelets/µL had a lower capacity to be activated, as shown through the reduced binding of PAC1 to fibrinogen receptors (Figure 3A) and the diminished exposure of P-selectin and CD63, released, respectively, from alpha and dense granules (Figure 3B,C). This impairment in the platelets’ stimulation capacity was not due to a reduced content of fibrinogen receptor (Figure 3D). Moreover, platelets from ITP patients also exposed similar levels of VWF receptor on their surface (Figure 3D).

The patients with the lowest platelet count showed the most pronounced decrease in platelet activation markers induced by TRAP stimulation (platelet count vs. PAC1 binding: *ρ* = 0.392, *p* <0.001; vs. P-selectin *ρ* = 0.451, *p* < 0.001; and vs. CD63 exposure *ρ* = 0.229, *p* < 0.001).

### 3.4. Apoptosis Markers of Platelets

**Figure 4:**
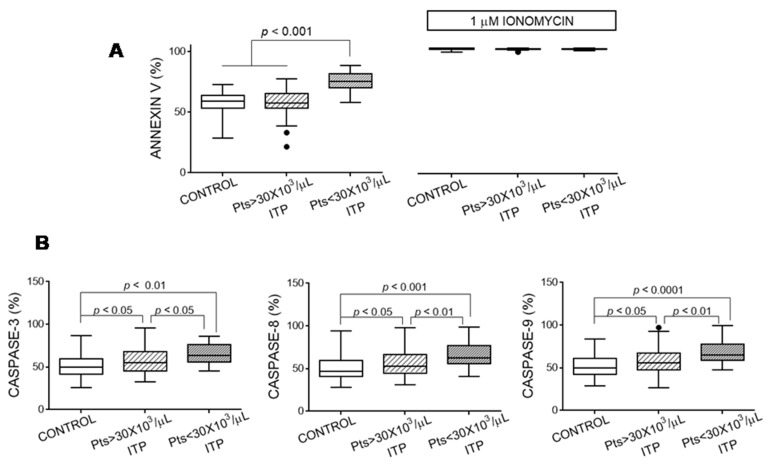
Apoptosis signs of platelets. (**A**) Phosphatidylserine surface exposure determined by binding of fluorescein isothiocyanate (FITC)-annexin V in platelets incubated with either buffer or ionomycin, and (**B**) caspase activities in quiescent platelets from controls and patients with immune thrombocytopaenia (ITP) were determined by flow cytometry analysis. Data are expressed as percentage of positive cells. We performed Kruskal–Wallis and Dunn’s multiple comparison tests and considered a *p*-value < 0.05 as significant.

Platelets from the patients with ITP and a platelet count <30 × 10^3^/µL showed more pronounced signs of apoptosis (Figure 4). In another cohort of patients with ITP, we previously reported an inverse relationship between platelet apoptosis and the platelets’ ability to be activated by agonists [4]. We confirmed that this association was also true in individuals included in the present study (TRAP-induced PAC binding vs. caspase 3: *ρ* = −0.262, *p* < 0.001; vs. caspase 8: *ρ* = −0.301, *p* < 0.001; and vs. caspase 9: *ρ* = −0.228, *p* < 0.01).

### 3.5. Relationship between Glycosylation and Platelet Functional Characteristics

**Figure 5:**
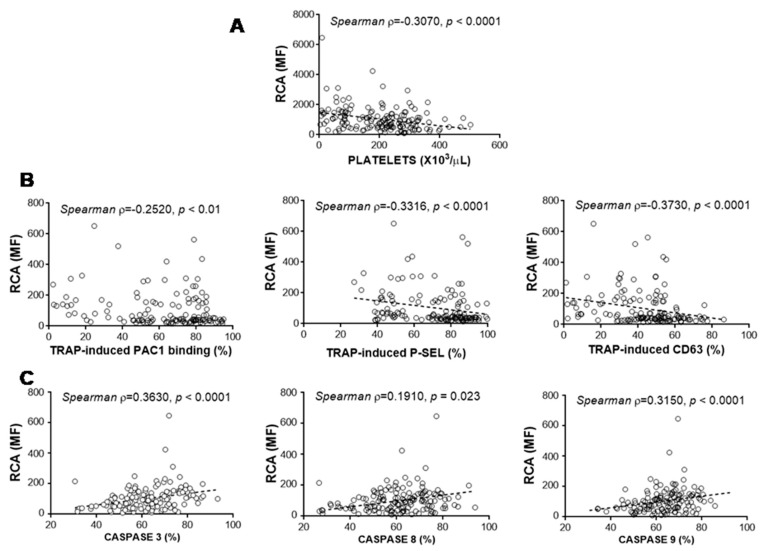
Loss of sialic acid was indirectly determined through the binding of Ricinus communis agglutinin (RCA). Correlation between RCA binding and platelet count (**A**), platelet activation markers (**B**), and caspase activities in quiescent platelets (**C**) was determined by Spearman’s test, and a *p*-value < 0.05 was considered significant.

We studied whether there was a relationship between glycosylation on the platelet surface and platelet functional features. We observed that the platelet count and the ability to be stimulated were related to glycoside residues on the platelet surface (Table 3). Particularly, the loss of sialic acid residues (measured indirectly through the RCA binding) appeared to be a key player in reducing platelet counts (Figure 5A) and the platelets’ ability to be activated (Figure 5B). Moreover, lower sialic acid exposure on the platelet surface corresponded to higher activity of platelet caspases (Figure 5C).

### 3.6. Neuraminidase Activity in Plasma and Serum

**Figure 6:**
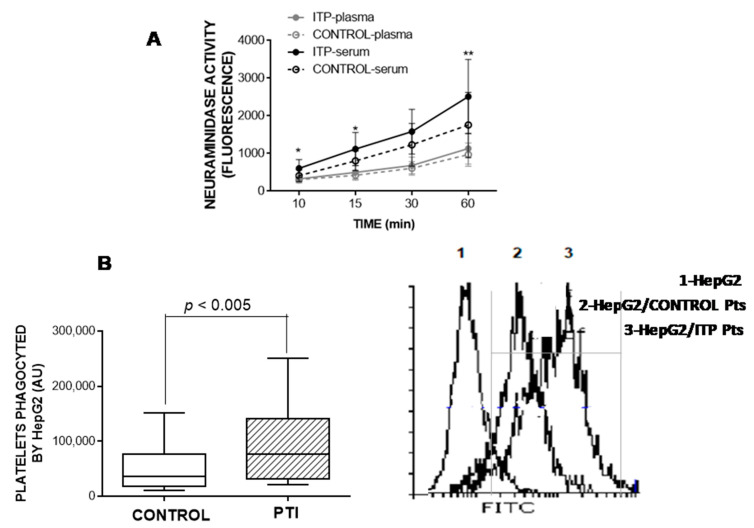
(**A**) Neuraminidase activity was measured in either serum (black lines) or plasma (grey lines) from healthy controls (open circles) and patients with immune thrombocytopaenia (closed circles), according to the Methods section. (**B**) HepG2 cell ingestion of human platelets in vitro. Ingestion of CMFDA-labelled platelets was detected by flow cytometry as an increase in hepatocyte-associated fluorescence (*y*-axis, in arbitrary units, AU). Representative flow cytometry histograms are shown. Mann–Whitney test was performed to determine the differences between the control and immune thrombocytopaenia groups, and a *p*-value < 0.05 was considered significant.

Neuraminidase is an enzyme that mediates the release of sialic acid. We therefore measured its activity and relationship with RCA binding and the platelets’ capacity to be activated. Neuraminidase activity measured over time was higher in the serum from the patients with ITP than in the serum from the healthy controls, whereas there was no difference in plasma neuraminidase activity between these groups (Figure 6A). As expected, we found a direct relationship between neuraminidase activity in serum and RCA binding to platelets (*ρ* = 0.7030, *p* < 0.001).

Supporting the involvement of sialic acid in platelet activation capacity, we observed an inverse correlation between neuraminidase activity in serum and TRAP-induced PAC binding (*ρ* = −0.453, *p* < 0.05).

### 3.7. HepG2-Based Platelet Ingestion Assay

We determined the degree of labelled platelets ingested by HepG2 cells by flow cytometry. As shown in Figure 6B, HepG2 cells ingested more platelets isolated from the patients with ITP than platelets from the healthy control group. The lower exposure of sialic acid induced the highest ingestion of platelets (*ρ* = 0.5510, *p* < 0.01).

## 4. Discussion

Our results revealed a close relationship between ITP severity (evaluated through platelet counts and the platelets’ ability to be activated) and loss of sialic acid from glycoproteins on the platelets’ surface. The three major glycosylated proteins in the platelet glycocalyx are P-selectin (13,000 copies per activated platelet), the GPI-IX complex (CD42a/CD42b, 25,000 copies per platelet), and the integrin GPIIbIIIa (CD41/CD61; 50,000 copies per platelet) [17].

Loss of sialic acid is responsible for increasing platelet clearance through the hepatic Ashwell–Morell receptors present in liver cells [18]. Along these lines, we demonstrated that platelets with the lowest exposure of sialic acid were the most ingested by human hepatome HepG2 cells. Desialylation of platelet glycoproteins might be due to their sialidase activity, which relies on 4 sialidases (NEU1-NEU4) that are found at different locations and have different affinities for their substrates [19,20]. NEU1, NEU2, and NEU4 are present on the surface of quiescent platelets. Exposure of NEU1 and NEU2 on the platelet surface increased after specific clustering of GPIb activated by VWF, which mobilised them from their intracellular stores (mitochondria for NEU1 and α-granules for NEU2) [16]. Another situation that upregulates NEU1 on the platelet surface is the presence of anti-GPIbα antibodies, such as those present in some patients with ITP [18,21]. Our data showed that platelets from most of our patients with ITP had less sialic acid than the platelets from the healthy controls, despite the fact that anti-GPIb antibodies were detected only in one of the patients whose platelets did not have any singular feature when compared with those from the patients with ITP with similar platelet counts (134 × 10^3^/µL). Moreover, the agonists employed in our experiments to stimulate platelets did not induce NEU1 and NEU2 expression on the platelet surface [16]. We also observed that serum (but not plasma) from the patients with ITP had an increased neuraminidase activity, suggesting a cellular source for the enzyme. Taken together, our data suggest that other mechanisms underlying the changes in platelet surface glycosylation must be at play. For example, it has been reported that CD8+ T cells from patients with ITP with positive cytotoxicity induced significant platelet desialylation, NEU1 expression on the platelet surface, and platelet phagocytosis by hepatocytes in vitro [22].

Studies have reported that desialylated GPIba has an increased ability to be activated by ristocetin and to bind to VWF [23,24]. Moreover, a study elegantly demonstrated that NEU1 and NEU2 are specifically translocated to the membrane following VWF-mediated GPIba-clustering and that this event produces desialylation that potentiates the ability of αIIbβ3 to bind to fibrinogen [25]. This observation does not contradict our results (a diminished activation of fibrinogen receptor due to the loss of sialic acid) because we specifically tested platelet activation through the inside-out stimulation of fibrinogen receptor with no involvement of the VWF receptor. Another example of thrombocytopaenia due to desialylation induced by excessive binding of VWF to platelets is that observed in patients infected by the dengue virus [26]. Further supporting our results, the treatment of platelets with neuraminidase reduced their aggregation induced by ADP [27]. Moreover, abnormalities in the N-glycosylation of GPVI could contribute to acquired defects in GPVI-mediated platelet reactivity to collagen [8,28,29].

The function of platelet receptors that mediate their adhesive and aggregative properties are strongly reliant upon N-glycosylation to modulate the orientation of glycoproteins to facilitate interaction between proteins [29]. A mutational study performed on β3 demonstrated that loss of N-glycoside sites impaired either αIIbβ3 expression or function. The first possibility does not seem to explain our results because we did not detect a decrease in the surface expression of fibrinogen receptor [30]. Therefore, the platelets’ diminished function might be explained by the fact that many of these N-glycan sites lie in the domain interfaces that are rearranged during integrin activation.

Another cause for the reduced exposure of sialic acid on the platelet surface might be a decrease in glycosyltransferase activity. Three distinct glycosyltransferase families were found within and on the surface of platelets [31]. Supporting the importance of sialyl transferases in the platelets’ lifespan, mice lacking ST3GAL4 (an enzyme that transfers sialic acid onto β1,4-galactose) develop thrombocytopaenia due to increased platelet clearance via the hepatic Ashwell–Morell receptor [32]. Moreover, the lack of ST3GAL1 (an enzyme that caps the Thomsen–Friedenreich antigen with sialic acid) was associated with platelet counts 50% lower than normal, as observed in ST3GAL1-null mice [33,34].

Platelets from ITP patients showed more signs of apoptosis. The percentage of resting control platelets positive for annexin V maybe higher than those observed by other authors, but it has been reported that variations in experimental conditions may explain these differences [35]. We found a direct correlation between the loss of sialic acid and the activity of platelet caspases 3, 8, and 9 (present results and those of Monzon Manzano et al. [4]). Moreover, it has been reported that ABT-737 (an inhibitor of Bcl-2 family proteins) induced apoptosis of dog platelets, with the primary platelet clearance site being the liver [10]. Nevertheless, Grodzielski et al. proposed that platelet apoptosis and loss of sialic acid were not necessarily related [36].

Desialylation was the main focus of this study. However, we also detected changes in the distribution of other glycoside residues in the platelets from the patients with ITP, as well as an inverse correlation between α1,6-fucose, α-mannose, GalNAc, and β-GlcNAc exposure and platelet count and activation capacity of the fibrinogen receptor. Enhanced exposure of β-GlcNAc was revealed through the increased binding of WGA to platelets from the patients with ITP and might indicate another mechanism of platelet destruction given that β-GlcNAc residues can be recognised by the α_M_β2 hepatic macrophage receptors and phagocytosed by these cells [37,38], as was reported for cold-storage platelets [11]. This mechanism for platelet clearance seemed to be independent of that mediated by Ashwell–Morell receptors, because HepG2 cells express both chains (ASGR1 and ASGR2) of the Ashwell–Morell receptor but do not express α_M_β2. Moreover, Rumjantseva et al. [11] demonstrated that macrophage-mediated clearance was operative for platelets chilled for 4 h, whereas refrigeration for a longer period (24 h) induced the removal of platelets through hepatic Ashwell–Morell receptors.

Changes in glycoside composition on the surface of platelets from patients with ITP might also have consequences for complement activation. Collectins (e.g.,mannose-binding lectin) and ficolins are pattern-recognising molecules that are not only reactive against pathogen-associated molecular patterns but also to aberrantly glycosylated self cell-surface structures [39]. These lectin pathway-related pattern-recognising molecules of the complement recognise residues of carbohydrates such as D-mannose, GlcNAc, and L-fucose, all of which are increased on the surface of platelets from patients with ITP. Moreover, mannose-binding lectin binds to platelets [40], and sialic acid residues inhibit its binding to cells [41]. It is therefore tempting to speculate that a complement is involved in the etiopathogenesis of ITP, as proposed by numerous authors [42,43].

## 5. Conclusions

Our data suggest the importance of glycoside residues present on the surface of platelets for determining their count and functionality in patients with ITP. Moreover, the results of this study encourage future studies to further elucidate the participation of the platelet glycome in the etiopathogenesis of ITP, given that the presence of sialic acid is a good sensor for the discrimination of “self” and “non-self” signals to regulate the innate and adaptive immune system responses [44]. In further support of the glycome in the immune response, we have previously observed an inverse correlation between loss of sialic acid and LTreg counts in patients with ITP [4]. Failure of the immune system to correctly distinguish “self” is one hallmark of autoimmunity [45,46].

## Figures and Tables

**Table 1:** Lectins used in flow cytometry experiments and their glycoside binding specificity.

Lectin	Aleuria Aurantia	Concavalin A	Datura Stramonium	Ricinus Communis Agglutinin I	Wheat Germ Agglutinin
Abbreviation	AA	C	DS	RCA	WGA
Sugar specificity	α1,6-Fucose	α-Mannose	GalNAc	Galactose GalNAc	β-GlcNAc

GalNAc: N-acetylgalactosamine; β-GlcNAc: N-acetylglucosamine.

**Table 2:** Therapeutic treatments of patients with immune thrombocytopaenia (ITP).

	<30,000 Platelets/µL			>30,000 Platelets/µL		
	Gender (%)	Age Mean ± SD	Concomitant Medication (Nº Patients)	Gender (%)	Age Mean ± SD	Concomitant Medication (Nº Patients)
No treatment	M: 2 (50) F: 2 (50)	49 ± 21.6	-	M: 11(34) F: 21 (66)	56 ± 19	+Anticoagulant: 3
Eltrombopag	M: 3 (50) F: 3 (50)	46 ± 11.8	+IGIV (2) +Corticosteroids (1) +Corticosteroids + azathioprine (2)	M: 3 (21) F: 11 (79)	54 ± 21.5	+IGIV: 1 +Anticoagulant: 5
Romiplostim	M: 3(50) F: 3(50)	58 ± 25.9	-	M: 9 (56) F: 7 (44)	57 ± 19.5	+IGIV: 1 +Corticosteroids: 5
IGIV	M: 0 (0) F: 1(100)	62	-	-	-	-
Corticosteroids	-	-	-	M: 0 F: 3(100)	51 ± 2.5	+IGIV: 2 +Rituximab: 1

IGIV: intravenous immunoglobulins, M: male, F: female, Nº: the number of patients.

**Table 3:** Correlation between glycoside exposure and platelet count and ability of fibrinogen receptor to be activated.

Recognised Glycoside Residue (Lectin)	α-1,6 Fucose (AA)	α-Mannose (C)	GalNAc (DS)	β-GluNAc (WGA)
Lectin binding vs. Platelet count (Spearman *ρ*, *p*)	−0.3946; 0.0002	−0.3964; 0.0002	−0.4091; 0.0001	−0.2868; 0.0177
Lectin binding vs. TRAP-PAC1 binding (Spearman *ρ*, *p*)	−0.2297; 0.392	−0.2567; 0.040	−0.2581; 0.0185	−0.3737; 0.0007

AA: Aleuria aurantia; C: Concanavalin A; DS: Datura stramonium, GalNAc: N-acetylgalactosamine; WGA: Wheat germ agglutinin; β-GlcNAc: N-acetylglucosamine. Correlation was determined by Spearman’s test, and a *p*-value < 0.05 was considered significant.

## Data Availability

Data is contained within the article.
